# *Code Red for Health* response in Latin America and the Caribbean: Enhancing peoples' health through climate action

**DOI:** 10.1016/j.lana.2022.100248

**Published:** 2022-04-20

**Authors:** Marisol Yglesias-González, Yasna Palmeiro-Silva, Milena Sergeeva, Sandra Cortés, Andrea Hurtado-Epstein, Daniel F. Buss, Stella M. Hartinger, Red de Clima y Salud de América Latina y el Caribe

**Affiliations:** a Latin American Centre of Excellence for Climate Change and Health, Universidad Peruana Cayetano Heredia, Av. Honorio Delgado 430, San Martín de Porres, Lima 15102, Peru; b Institute for Global Health, University College London, UK; c Centro de Políticas Públicas UC, Pontificia Universidad Católica de Chile, Chile; d Liaison Officer for LAC, Global Climate and Health Alliance, USA; e Department of Public Health, School of Medicine, Advanced Center for Chronic Diseases, Centro de Desarrollo Urbano Sustentable, Pontificia Universidad Católica de Chile, Chile; f Climate Program Manager for Latin America, Health Care Without Harm, USA; g Pan American Health Organization (PAHO), Washington, DC, USA

**Keywords:** Climate change, Greenhouse effect, Population health, Latin America, Caribbean region

## Introduction

The COVID-19 pandemic has affected peoples’ lives in Latin America and the Caribbean (LAC), exacerbated social inequities, revealed deficient health systems,^1^ and triggered an economic recession^2^ that is already pushing a significant proportion of people into poverty.^3^ However, this current situation pales in comparison to the impacts that human-induced climate change is having and will have on people's lives and livelihoods. Climate change will magnify the health hazards many face, particularly populations exposed to food and water insecurity, heatwaves, and infectious diseases.^4^

The Intergovernmental Panel on Climate Change (IPCC) clearly confirms that the climate in Central and South America has changed and projects more extreme weather patterns, glacier volume loss, and sea-level rise.^5^ On the other hand, Caribbean populations are in the front line of the impacts of climate change, experiencing record temperatures, widespread droughts, abundant tropical waves, intence rainfalls and hurricanes.^28^ Additionally, the bleaching and damage of coral reefs puts at stake marine life and threatens the livelihoods of LAC, but particularly, of Caribbean community.^35^

Similarly to the inequitable response that the world is delivering to the pandemic, there are profound differences in the global response to climate change. The latest *Lancet* Countdown report urges world leaders to commit to urgent action to address the negative trends of the health impacts of climate change. Lack of improvement across the 44 indicators that the annual report tracks show how in the past five years, globally, all indicators are worsening.^4^ This highlights the threat of climate change to the stability of the Earth system and humanity, pushing the limits of a safe operating space and increasing the risk of irreversible global environmental changes.^6^

LAC countries emit relatively small amounts of greenhouse gases (GHG),^7^^,^^8^ and yet, most fail to comply with GHG emission targets in their Nationally Determined Contributions (NDCs) to reach the Paris Agreement central aims.^9^ Governmental policies and responses to protect peoples’ health from climate change with health-inclusive and health-promoting climate targets vary significantly in LAC. The analysis done by the Global Climate and Health Alliance (GCHA)^10^ to determine if the existing NDCs commitments are enough to protect health, shows that countries like Argentina, Costa Rica, and Colombia included health as a relevant factor in terms of climate impacts and health co-benefits, thus expressing that protecting the health of the populations is a priority, while maximising economic benefits, and ensuring wider public support for ambitious climate policies. The Dominican Republic and Belize are also successful examples of multisectoral collaboration in the development of the NDCs with the active participation from their Ministries of Health. Both countries have included health as one of the cross-sectoral mitigation and adaptation priorities.^11^^,^^12^ However, some countries that consider health extensively their national commitment have, at the same time, failed to set emission reduction targets that align with the Paris Agreement and with health protection.^10^

Despite the increasing evidence on the health impacts of climate change, LAC countries are still not delivering a response proportionate to the rising risks. Although health was recognized as a priority topic by 83% of NDCs from LAC, most NDCs do not commit with specific health actions translating to less than 0.5% of multilateral climate finance for LAC countries allocated to health initiatives.^13^^,^^14^ For instance, Brazil's actions alone are crucial for global climate outcomes, as the country hosts a significant portion of one of the world's major carbon sinks: the Amazon rainforest, which is now flipping to a carbon source due to deforestation and climate change.^15^^,^^16^ Brazil reduced its climate ambition in 2020 regarding its NDCs mitigation strategies.^17^, ^18^, ^19^

In this viewpoint, we echo the *“Code Red for Health”* call from the 2021 *Lancet* Countdown report,^4^ discuss the overlapping social, climate and health challenges in LAC, and urge action on the different pathways to transform these challenges into opportunities through adaptation and mitigation measures that place peoples’ health and wellbeing at the centre of public policies. We also call for LAC governments to promote climate-resilient health care systems with adaptation plans that are tailored to guarantee quality access to care for all.^20^ Bold, fast, and equitable climate action is urgently needed to protect people's health and wellbeing in the LAC region.

**Box 1. The Climate and Health Network of Latin America and the Caribbean**

**The Climate and Health Network of Latin America and the Caribbean^¥^**

The Climate and Health Network of Latin America and the Caribbean is convened by the Global Climate and Health Alliance and includes health professional and health student organisations, climate and health research centres, and NGOs that work to minimise the impacts of climate change on human health and to obtain benefits for public health from climate mitigation and adaptation. The network represents a wide diversity in geography and expertise in LAC.

*Vision*

The organisations comprising the Climate and Health Network of Latin America and the Caribbean share the vision of a healthy, sustainable, equitable, resilient, and inclusive Latin American and the Caribbean region, leading in public health-centred climate and development policies, leaving no one behind, and enjoying quality and affordable healthcare.

*Mission*

The network intends to collaborate with national governments, multilateral and non-governmental organisations using scientific evidence in awareness-raising, advocacy, and decision-making in climate action and health throughout Latin America and the Caribbean, developing recommendations for public policies by virtue of the protection of public health, equity, and climate justice.

Alt-text: Unlabelled box

## Overlapping social, climate and health challenges in LAC

LAC is a territory that encompasses 33 countries, rich in cultures, landscapes, biodiversity, ethnicities, and bioclimates. It is also the home of 40 million indigenous people, who in vast majority depend on fragile livelihoods threatened by a changing climate,^21^ while in many cases living on and stewarding ecosystems rich in biodiversity.^22^ Unfortunately, due to social and economic inequality, and low investment in public health, adverse health outcomes and health inequities are still important challenges in the region. Adding to this, we must consider two additional challenges: political stability and policy continuity and climate funding. For the past decades, LAC countries continue to experience ongoing political turmoil with increasing citizen dissatisfaction towards the inaction of political elite; increased perception of corruption^23^; and lack of environmental governance.^24^

Considering that climate change is deepening existing inequalities,^4^ and disproportionally affects disadvantaged populations, having underfunded health systems can make countries more vulnerable to the new challenges posed by climate change. For LAC countries, this translates into even more likely deficient health service delivery and infrastructure, putting public health and the achievement of the Sustainable Development Goals (SDGs) at risk. Constrasting numbers are seen within and between LAC countries, and their health sectors capacity, preparedness, responce, recovery and resource allocation.

For example, health expenditure has grown in LAC,^25^ but the distribution is highly unequal. While countries like Argentina, Brazil, Chile, Cuba, and Uruguay spend over 9% of their GDP on health, Venezuela spends less than 4%.^26^ Furthermore, the overall growth in expenditure has not necessarily translated into more sustainable and resilient health systems, and even where there are deliberate efforts to promote such a transition, the resources allocated to it greatly vary. For example, while Uruguay spent US$5.61 per capita on climate adaptation for health and health-related activities in 2019-2020, Honduras spent US$0.71.^4^ These numbers show the disparities within LAC in its capacity to respond and adapt to a changing climate, placing many countries at a disadvantaged starting point.

The LAC region is a clear example of the increasing inequality between the geography of emissions and the geography of impacts. While in 2018 LAC countries generated 2.6 metric tons of carbon dioxide (CO_2_) emission per capita, high income countries emitted 10.3 metric tons of CO_2_ emissions.^27^ Meanwhile, between 1998 and 2020, climate events have affected 277 million people and resulted in the deaths of over 300,000 people in the LAC region.^28^

In terms of climatic hazards, the State of the Climate in Latin America and the Caribbean report from the World Meteorological Organisation^28^ and the AR6 WGI IPCC report^29^^,^^30^ show current and future changes in the climate system in comparison to preindustrial levels. These changes include extreme tropical cyclones, storms, and dust storms; severe and expanded droughts; increasing trends of mean temperature at greater rates than the global average leading to glacier loss and heatwaves; variations in mean precipitation that can be accelerated by current regional land use change and deforestation rates; continuity on relative sea-level rise; and an increase in marine heatwaves.

The 2021 *Lancet* Countdown report sends a clear message of alert with a *“Code Red for Health”*, highlighting an increased suitability for the transmission of dengue, zika and chikungunya from the *Aedes aegypti* in Brazil and Peru.^4^ Dengue cases have almost tripled from 2000-2009 (6.78 million) to 2010-2019 (16.52 million) and the largest record of cases occurred in 2019.^31^^,^^32^ Furthermore, exposure to heat waves has also increased in the region, reaching almost 270 million person-days in vulnerable populations (>65 years) in 2020.^4^ It is estimated that between 20% and 60% of heat-related mortality in South America was attributed to anthropogenic climate change between 1991 and 2018.^33^ Finally, excessive red meat consumption has three upshots. Land-use change for cattle production, emissions of GHG, especially methane, and an increase in premature mortality. The *Lancet* Countdown reports a staggering 66,000 premature and preventable deaths attributable to red meat consumption in 2018 in Argentina, Brazil and Colombia alone.^4^

Climate change can lead to forced displacement, migration, and population mobility, along with the life losses and economic impact that it entails. The economic impacts of extreme weather events in relation to the size of the economy is singular for the Caribbean region,^34^ added to a significant threat to sea level rise. It has been estimated that the economic cost of Hurricane Maria in Dominica, was 260% of its annual GDP, along with a displacement of up to 27.3% of its total population.^34^ Thousands of people from these islands are also at risk of experiencing future sea level rise. 100 thousand people in Haiti alone, are at risk of 1 m future sea level rise by the end of the century according to projected estimates.^4^

## Transforming challenges into opportunities to improve peoples’ health

The aforementioned challenges share many human-induced drivers, which can be transformed into opportunities. LAC countries can choose to respond to the current critical juncture with a comprehensive vision and, through enhanced intersectoral policy coherence, to simultaneously promote better public health systems, economic actions that target reduction of inequalities, protect the environment and ecosystem services, manage climate risks, and ultimately, improve peoples’ health. Climate action, both mitigation and adaptation, must be at the centre of a broad and profound transition towards more inclusive, just, and sustainable development models, particularly considering that the economic health gains of tackling climate change substantially outweigh the costs of achieving them.^14^

LAC countries are heterogeneous in climate, ecosystems, human population and culture, but they share similar vulnerabilities, reflected in low adaptive capacity, especially of health systems.^14^ A territorial approach to community health risks that puts a focus on gender, equity, and vulnerable populations must be the priority for resilience building and adaptation.^36^ The latest report from *Lancet* Countdown shows that LAC cities are making efforts to assess the climate change risks faced at city level. Out of 250 surveyed cities in LAC, a total of 182 have a climate change vulnerability and risk assessment plan in progress or done.^4^ This reflects an important step towards climate change adaptation. Duly implementing tailored plans based on specific vulnerabilities associated with social and environmental characteristics of each territory, while incorporating health systems offers an immense opportunity to protect people's health.

Complementarily, the success of climate mitigation is key as it can provide health co-benefits at local level. Considering that emissions are projected to increase and that up to 15% of COVID-19 mortality rates have been attributed to long-term exposure to air pollution,^37^ a decarbonised economy is essential to reach the Paris Agreement targets and improve health. Some decarbonization efforts in the region are commendable. Chile is the first country in the region to introduce a carbon tax^4^ and its electricity generation from low-carbon source emissions has increased in the past years, from 0% in 2007 to 14% in 2019, placing Chile above the OECD countries’ average.^38^ Moreover, Mexico is ranked within the top 50 countries of the world with the highest production of electricity from renewable sources.^4^ Policies linked to sustainable transportation and phasing out fossil fuel subsidies could help build cleaner cities and transportation systems that encourage physical activity, and improve health by reducing exposure to air pollution.

Green spaces in cities sequester carbon and provide local cooling that reduces urban heat islands, benefiting both climate change mitigation and heat adaptation. According to the 2021 *Lancet* Countdown report, there are fewer cities in LAC than the rest of the world (27%) with green urban space. For example, Lima is one of the 6 capitals with the least green space in the world.^4^ Investing in green spaces is a great opportunity for LAC cities as it reduces air and noise pollution, reduces the heat island effect, relieves stress, promotes physical activity, and reduces all-cause mortality.^4^^,^^38^

Further opportunities to promote good health for all while decreasing GHG emissions involve advancing healthy, sustainable, and equitable food systems. Brazil's National School Feeding Program (Programa Nacional de Alimentação Escolar; PNAE) is an innovative sustainable policy, that within the framework of Brazil-FAO International Cooperation Program,^39^ stipulates by law that a minimum quota of 30% of PNAEs funds is to be spent in procurement of organic products from local family farms. This initiative not only provides access to a healthier diet, but also promotes sustainable agriculture, fosters local economic growth, and reduces GHG emissions from the food supply chain.^40^

All the above can only be achieved by mobilising a significant amount of climate finance, which is essential to implement mitigation and adaptation plans. The Addis Ababa Action Agenda on Financing for Development posits that domestic public resources, supplemented by international assistance as appropriate, are critical to realising sustainable development.^41^ In that context, LAC countries must be responsible for the implementation of their own NDCs and NAPs, through tangible and transparent financial commitments that translate to real budget allocations, and for putting in place monitoring and evaluation mechanisms that allow for accountability and the assessment of progress.

However, LAC countries will require significant international support if they are to adopt truly ambitious and transformative climate targets and plans. It is worth noting that countries in the region, along with other low- and middle-income countries around the world, have been subjected to a longstanding broken promise of funding from industrialised nations.^42^ As stated in the Paris Agreement and mandated under Article 9,^43^ industrialised countries must mobilise USD $100 billion per year to finance climate action in the Global South, a promise they are yet to deliver on. In addition, they must promote endeavours to make international financial mechanisms more accessible to developing countries.^42^

For example, the Green Climate Fund (GCF) and Global Environmental Facility (GEF) have highly complex requirements that make their resources notably difficult to access, which most LAC countries lack the capacity to tackle (to illustrate, as of October 2021, only 17 institutions in the region operate as accredited entities to implement GCF projects).^44^ In addition, access to international finance is especially challenging for LAC countries because most of them are categorised as middle-income (a classification that is solely based on GDP), and they are therefore not prioritised for international cooperation, while those who have “graduated” are no longer eligible to receive official development assistance.^45^ This is further complicated by the extreme debt crisis submerging many countries in the region, particularly in the Caribbean.

In this context, it is more crucial than ever that developed countries support international initiatives that aim to simultaneously address the health and climate crises, such as the COP26 Health Programme,^46^ and fully fund action to prevent and address loss and damage from climate impacts, particularly through the Warsaw International Mechanism and the Santiago Network on Loss and Damage.^47^ For LAC to be in a position to respond to mutually reinforcing climate and health risks in a decisive and transformative manner, it is essential to not only secure the means of implementation, but to facilitate effective regional access to them. Nonetheless, in the face of the converging crisis of the COVID-19 pandemic and the climate emergency, – and to understand the climate and environmental drivers of emerging and re-emerging diseases, to be able to anticipate, prevent, prepare, respond, and recover – all countries must act regardless of their level of development, and the larger economies of the LAC region are called upon to lead by example. This has, unfortunately, not been the case so far. According to the UN green recovery programme, environmentally sustainable rescue and recovery spending in LAC is imperceptible.^48^ Countries should seize the opportunity to structure their COVID-19 economic recovery around environmentally sound measures that build a more sustainable future. This can bring a multiplier effect with the creation of green jobs, the reduction of emissions, the increase in resilience, and ultimately, economic growth.^49^

## Health sector as a key actor in climate action

Health professionals have a trusted voice that can influence peoples’ health and wellbeing in the context of the climate crisis.^50^^,^^51^ The health sector becomes a key actor when it comes to advocacy, articulating the need for climate action, and bridging to other actors, favouring intersectoral action through the lens of health co-benefits. Health professionals are increasingly engaged on the issue-from working with communities impacted by climate change, to taking on climate advocacy and working to reduce emissions from health systems.^50^^,^^52^ Health professionals and climate scientists are trusted voices in most societies and can therefore be champions to help design climate policies that improve health outcomes and human well-being.

Health systems have a significant environmental footprint. Organisations such as Health Care Without Harm estimated that the sector's carbon footprint in 2014 represented 4.4% of global net GHG emissions,^53^^,^^54^ with an increase up to 4.9% according to the latest *Lancet* Countdown report.^4^ Climate resilient and environmentally sustainable health care facilities contribute to a high quality of care and accessibility of services, and by helping reduce facility costs, also ensure better affordability.^55^^,^^56^ The health sector can strengthen the overall health system and improve health care facilities, which are the first line of defence in case of climate-related extremes and shocks.^55^ Building climate-resilient health systems has become paramount to any national adaptation strategy, especially through adaptation measures that simultaneously boost health equity. Existing tools that assess vulnerabilities in health facilities,^57^ calculate and monitor carbon footprint,^58^ and guidance documents to build climate resilient health systems^59^ can support the sector to be better prepared in the context of climate change.

Governments can also achieve both health resilience and SDGs by (*i*) investing in health-determining sectors; (*ii*) prioritising the achievement of Universal Health Coverage and healthy lives and well-being (SDG3) as an overarching policy goal; (*iii*) ensuring coordination across sectors through interministerial committees on climate change, which includes the health sector; and (*iv*) monitoring synergies and trade-offs from actions in health-determining sectors,^55^ such as the energy, transport, food, and housing sectors. One example on how health systems can better adapt to climate change and build resilience is with heat wave early warning systems where national meteorological services are articulated with health systems. According to the 2021 *Lancet* Countdown report, only five out of 179 responding WHO member countries indicated that national climate services guide their policy and investment plans in the health sector; unsurprisingly, none are from LAC.^4^

One way to ensure that there is policy coherence for climate and health is to guarantee an explicit and high-level mandate for integrated implementation with monitoring and evaluation mechanisms. A key standard is including health in NDCs. For example, Costa Rica's NDCs is the only one in the region that aligns with the Paris Agreement.^60^^,^^61^ It acknowledges climate change impacts on health: vector-borne diseases and food security to name a few and commits to strengthen the knowledge, monitoring capacity and its health surveillance response services.^62^ Health is considered in adaptation and finance, and emphasis is given to the health benefits of specific mitigation goals with time limits in the transport and energy sectors. Another example is Argentina, which was the first country to include healthcare sector mitigation measures in its NDCs, and has included health as a “guiding axis” underpinning much of the content of its NDCs.^63^ Additional suggestions and examples on how to develop health-inclusive NDCs have been developed by the Pan American Health Organization/WHO.^64^ Moreover, this high-level mandate can also be adopted by health systems and facilities. For example, nineteen hospitals and medical institutions from the region have joined the health component of the UN Race to Zero campaign.^65^

The health sector is even becoming an actor in the international climate negotiations, as was made apparent by the unprecedented engagement of health experts during the 26th UN Climate Change Conference at Glasgow (COP26). Over 50 countries around the world joined the COP26 Health Programme,^46^ which called on countries to present national commitments to develop *(i)* climate- resilient health systems and *(ii)* sustainable, low emissions health systems.^66^ Signatory countries from LAC included Argentina, Bahamas, Belize, Chile, Colombia, Costa Rica, Jamaica, Panama, Peru, and Dominican Republic.^67^ These achievements could be interpreted as signs of progress.

Last year at COP, the UK Presidency organised a high level event focused on health, where the global health community highlighted the 10 recommendations of the WHO Special Report for COP, *The Health Argument for Climate Action*.^55^ In addition, 46 million health professionals addressed a Healthy Climate Prescription Letter^68^ to world leaders at COP26, pleading for health and equity to be put at the heart of climate response. The letter was formally delivered at the Presidency event and hundreds of health professionals from 12 LAC countries signed it in support of the global initiative. Panama reinforced the voice of the global health community in the plenary by expressing dissatisfaction with the fact that the COP26 final decisions did not recognise the importance of understanding and addressing the health impacts of climate change and the health benefits of climate action.^69^

## Conclusions

The current evidence shows that critical climatic trends are getting worse, exacerbating poverty and inequality due to delayed and inconsistent responses of countries around the globe; LAC countries are no exception. However, even within the LAC region and within each country, the populations least responsible for the situation will be the most affected. Furthermore, they will also be the last to enjoy the co-benefits of adaptation and accelerated decarbonization efforts if equity is not prioritized.

LAC governments need urgent political action to strengthen the health system and ensure that health is at the centre of the national and regional response to the climate crisis. The health sector should become the articulating crosswalk for other stakeholders and favour intersectoral action through the lens of health co-benefits. Countries must strengthen the health component of their climate policy instruments, particularly NDCs and National Adaptation Plans. In addition, LAC should draw from neighbouring countries' successful experiences to promote environmental and health governance.

Moreover, understanding of individual LAC countries' health-related vulnerabilities to climate change will support the identification of practical actions tailored to specific geographical difficulties to build resilient health systems. We must add to this complexity the additional challenge of training health professionals to be able to consider climate change impacts in their diagnosis and treatment plans. In most societies, they are a trusted voice which is present, as no other professional, in the most remote areas of LAC countries. Thus, the climate commitment should contemplate capacity development for health professionals, allocation of sufficient resources, and expansion of health services, as well as their monitoring and evaluation networks.

As citizens and as scientists, we call on governments to enhance climate ambition by reinforcing adaptation and mitigation measures, focusing on health and equity, and by accelerating phase out of fossil fuels while ensuring a just transition. From a space of science and advocacy, the *Climate and Health Network of Latin America and the Caribbean* offers research, assessment and advocacy collaboration to national governments, multilateral, and non-governmental organisations in harnessing the opportunity to improve the health and wellbeing of current and future generations.

## Contributors

MYG, MS, AHE and SH conceptualised the viewpoint; MYG, MS, AHE and YP wrote the first draft; SC, DB and SH performed critical revisions of the manuscript and contributed to the writing; All authors reviewed and agreed on the final version of the viewpoint. The Climate and Health Network of Latin America and the Caribbean agrees with the content of the viewpoint.

## Declaration of interests

The authors declare that there is no conflict of interest. DB is a staff member of the Pan American Health Organization (PAHO). The author alone is responsible for the views expressed in this publication, and they do not necessarily represent the decisions or policies of PAHO.

## Appendix Supplementary materials

Image, application 1

Image, application 2
