# Supplementary material for: Code Red for Health response in Latin America and the Caribbean: Enhancing peoples' health through climate action
Source: Lancet Reg Health Am. 2022 Apr 20;11:100248. doi: 10.1016/j.lana.2022.100248 (PMC9903933; doi:10.1016/j.lana.2022.100248)
Supplement: Supplementary file 1 [file mmc1.docx]

**Members list of the Climate and Health Network of Latin America and the Caribbean:**

|  | Nombre | Organización |
| --- | --- | --- |
| 1 | Dr. Mauricio Ilabaca | Departamento de medio Ambiente - Colegio Médico de Chile |
| 2 | Carolina Gil Posse | Salud Sin Daño |
| 3 | Antonella Risso | Salud Sin Daño |
| 4 | Doriam Camacho | Alliance of Nurses for Healthy Environments Latinoamérica |
| 5 | Dr. Paola Rava | A título personal |
| 6 | Esteban Arias | La Isla Network |
| 7 | Dr. Catalina Figueroa | Sociedad Chilena de Medicina del Estilo de Vida |
| 8 | Dr. Nelson Gouveia | Associação Brasileira de Saúde Coletiva |
| 9 | Ian Inestroza | IFMSA Honduras |
| 10 | Sofia Lasso | Grupo de trabajo Medicina y Medio Ambiente - Asociación de Sociedades Científicas de Estudiantes de Medicina de Colombia |
| 11 | Andrea Cortez | Federación Latinoamericana de Sociedades Científicas de Estudiantes de Medicina |
| 12 | Vital Ribeiro | Projeto Hospitais Saudáveis |
| 13 | Dr. Jeni Miller | Global Climate and Health Alliance |
| 14 | James Hospedales | EarthMedic/ EarthNurse |
| 15 | Luciana Blanco | Lancet Countdown Sudamérica |
| 16 | Dr. Damian Verzeñassi | Instituto de Salud Socioambiental UNR |
| 17 | Dr. Enrique Barros | Personal Signature |
| 18. | Dr. Nicole de Paula | Women Leaders for Planetary Health |
