# Supplementary material for: Code Red for Health response in Latin America and the Caribbean: Enhancing peoples' health through climate action
Source: Lancet Reg Health Am. 2022 Apr 20;11:100248. doi: 10.1016/j.lana.2022.100248 (PMC9903933; doi:10.1016/j.lana.2022.100248)
Supplement: Supplementary file 2 [file mmc2.pdf]

Editorial disclaimer: *This translation in Spanish was submitted by the authors and we reproduce it as supplied. It has not been peer reviewed. Our editorial processes have only been applied to the original manuscript in English, which should serve as reference.*

Artículo de opinión

Código Rojo para la Respuesta en Salud en América Latina y el Caribe: mejorando la salud de las personas a través de la acción climática

Marisol Yglesias-González, MScIH[a]; Yasna Palmeiro-Silva, MPH [b,c]; Milena Seergeva, MA[d]; Sandra Cortés, PhD [e]; Andrea Hurtado Epstein, MPhil [f]; Daniel Buss, PhD[f], Stella M. Hartinger, PhD.[a,*] y la *Red de Clima y Salud de América Latina y el Caribe*[¥].

Afiliaciones de todas las autoras:

[a] Centro Latinoamericano de Excelencia en Cambio Climático y Salud, Universidad Peruana Cayetano Heredia, Perú

[b] Instituto para la Salud Global, University College London, Reino Unido

[c] Centro de Políticas Públicas UC, Pontificia Universidad Católica de Chile, Chile

[d] Oficial de Enlace para ALC, Alianza Global para el Clima y la Salud, EE. UU.

[e] Departamento de Salud Pública. Escuela de Medicina. Centro Avanzado de Enfermedades Crónicas. Centro de Desarrollo Urbano Sustentable. Pontificia Universidad Católica de Chile, Chile

[f] Gerenta del programa de cambio climático para América Latina, Salud sin Daño, EE. UU.

[g] Organización Panamericana de la Salud (OPS), Washington, DC, EE. UU.

***Autora para correspondencia:** Stella Hartinger Peña, stella.hartinger.p@upch.pe, +51 987 113 911. Av. Honorio Delgado 430, San Martín de Porres 15102, Lima, Perú.

[¥] *La Red de Clima y Salud de América Latina y el Caribe.* Los miembros se enumeran en el Anexo.

Palabras clave: cambio climático, efecto invernadero, salud de la población, América Latina, región del Caribe

Recuento de palabras: 4719 (sin el texto en el Cuadro 1)

1. Introducción

La pandemia de COVID-19 ha afectado la vida de las personas en América Latina y el Caribe (ALC), ha exacerbado las desigualdades sociales, ha revelado sistemas de salud deficientes[1] y ha desencadenado una recesión económica[2] que ya está empujando a una proporción significativa de personas a la pobreza.[3] Sin embargo, esta situación actual pierde importancia en comparación con los impactos que el cambio climático inducido por el ser humano está teniendo y tendrá en la vida y subsistencia de las personas. El cambio climático magnificará los peligros para la salud que muchas personas enfrentan, en particular las poblaciones expuestas a inseguridad alimentaria y de agua, olas de calor y enfermedades infecciosas.[4]

El Grupo Intergubernamental de Expertos sobre el Cambio Climático (IPCC, por su sigla en inglés) confirma claramente que el clima en Centro y Sudamérica ha cambiado y proyecta patrones climáticos más extremos, pérdida de volumen de glaciares y aumento del nivel del mar.[5] Por otro lado, las poblaciones del Caribe están al frente de los impactos del cambio climático, experimentando temperaturas extremas, sequías generalizadas, abundantes olas tropicales, lluvias intensas y huracanes.[28] Además, el blanqueo y daño de los arrecifes de coral pone en juego la vida marina y amenaza los medios de subsistencia de los países de ALC y, en particular, de la comunidad caribeña.[3-5]

De manera similar a la respuesta desigual que el mundo está dando a la pandemia, existen profundas desigualdades en la respuesta global al cambio climático. El último informe de *Lancet* Countdown insta a los liderazgos mundiales a comprometerse con acciones urgentes para abordar las tendencias negativas de los impactos del cambio climático en la salud. La escasa mejora a lo largo de los 44 indicadores que el informe anual monitorea muestra cómo en los últimos cinco años, globalmente, todos los indicadores están empeorando.[4] Esto destaca la amenaza del cambio climático para la estabilidad del sistema terrestre y de la humanidad, empujando los límites de un espacio operativo seguro y aumentando el riesgo de cambios ambientales globales irreversibles.[6]

Los países de ALC emiten cantidades relativamente pequeñas de gases de efecto invernadero (GEI)[7,8] y, sin embargo, la mayoría no cumple con los objetivos de emisión de GEI que se encuentran en sus Contribuciones Determinadas a Nivel Nacional (NDC, por su sigla en inglés) para alcanzar los objetivos centrales del Acuerdo de París.[9] Las políticas y respuestas gubernamentales para proteger la salud de las personas del cambio climático con metas climáticas inclusivas y promotoras de la salud varían significativamente en ALC. El análisis realizado por la Alianza Global para el Clima y la Salud (GCHA, por su sigla en inglés)[10] para determinar si los compromisos de NDC existentes son suficientes para proteger la salud, muestra que países como Argentina, Costa Rica y Colombia incluyeron la salud como un factor relevante en términos de impactos climáticos y beneficios agregados para la salud, expresando así que la protección de la salud de las poblaciones es una prioridad, junto con la maximización de los beneficios económicos y el aseguramiento de un apoyo público más amplio para políticas climáticas ambiciosas. República Dominicana y Belice, con la participación activa de sus Ministerios de Salud, también son ejemplos exitosos de colaboración multisectorial en el desarrollo de las NDC. Ambos países han incluido la salud como una de las prioridades intersectoriales de mitigación y adaptación.[11,12] Sin embargo, algunos países que consideran

ampliamente la salud como su compromiso nacional han, al mismo tiempo, fallado en establecer metas de reducción de emisiones que se alineen con el Acuerdo de París y con la protección de la salud.[10]

A pesar de la creciente evidencia sobre los impactos del cambio climático en la salud, los países de ALC aún no están dando una respuesta proporcional a los crecientes riesgos. Aunque la salud fue reconocida como un tema prioritario en 83% de las NDC de ALC, la mayoría de las NDC no se comprometen con acciones específicas de salud, lo que se traduce, para los países de ALC, en menos del 0,5% del financiamiento climático multilateral asignado a iniciativas de salud.[13,14] Por ejemplo, las acciones de Brasil, por sí solas, son cruciales para los resultados climáticos globales, ya que el país alberga uno de los principales sumideros de carbono históricos del mundo: la selva amazónica, la cual se está convirtiendo ahora en una fuente de emisión de carbono debido a la deforestación y al cambio climático.[15,16] En 2020, Brasil redujo su ambición climática respecto a sus estrategias de mitigación de las NDC.[17–19]

En esta perspectiva, hacemos eco el llamado *"código rojo para la salud"* del informe 2021 de *Lancet* Countdown,[4] discutimos los desafíos sociales, climáticos y de salud superpuestos en ALC e instamos a la acción en las diferentes vías para transformar estos desafíos en oportunidades a través de medidas de adaptación y mitigación que coloquen la salud y el bienestar de las personas en el centro de las políticas públicas. También hacemos un llamado a los gobiernos de ALC para que promuevan sistemas de salud resilientes al clima con planes de adaptación diseñados para garantizar que todos tengan un acceso de calidad a la atención.[20] Se necesita con urgencia acción climática audaz, rápida y equitativa para proteger la salud y el bienestar de las personas en la región de ALC.

Cuadro 1. La Red de Clima y Salud de América Latina y el Caribe

La Red de Clima y Salud de América Latina y el Caribe[¥]

La Red de Clima y Salud de América Latina y el Caribe es convocada por la Alianza Global por el Clima y la Salud e incluye organizaciones de profesionales de la salud y estudiantes de la salud, centros de investigación sobre clima y salud y organizaciones no gubernamentales (ONG) que trabajan para minimizar los impactos del cambio climático en la salud humana y obtener beneficios para la salud pública a partir de la mitigación y adaptación climática. La Red representa una amplia diversidad en geografía y experiencia en ALC.

Visión

Las organizaciones que integran la Red de Clima y Salud de América Latina y el Caribe comparten la visión de una región de América Latina y el Caribe saludable, sostenible, equitativa, resiliente e inclusiva, que lidere en las políticas climáticas y de desarrollo centradas en la salud pública, sin dejar a nadie atrás, para disfrutar de una atención en salud asequible y de calidad.

Misión

La Red tiene como objetivo colaborar con gobiernos nacionales, organismos multilaterales y no gubernamentales utilizando evidencia científica en la sensibilización, incidencia y toma de decisiones

en acción climática y salud en toda América Latina y el Caribe, desarrollando recomendaciones de políticas públicas en virtud de la protección de la salud pública, la equidad y la justicia climática.

2. Superposición de desafíos sociales, climáticos y de salud en ALC

ALC es un territorio que abarca 33 países, ricos en culturas, paisajes, biodiversidad, etnias y bioclimas. También es el hogar de 40 millones de personas indígenas, que en su gran mayoría dependen de medios de subsistencia frágiles amenazados por un clima cambiante,[21] al mismo tiempo que, en muchos casos, viven y cuidan ecosistemas ricos en biodiversidad.[22] Desafortunadamente, debido a la desigualdad social y económica y a la baja inversión en salud pública, los resultados adversos en salud y las inequidades en salud siguen siendo desafíos importantes en la región. Además de esto, debemos considerar dos desafíos adicionales: la estabilidad política y continuidad de las políticas y el financiamiento climático. Durante las últimas décadas, los países de ALC continúan experimentando constante agitación política con una creciente insatisfacción de la ciudadanía por la inacción de la élite política; mayor percepción de corrupción;[23] y falta de gobernanza ambiental.[24]

Considerando que el cambio climático está profundizando las desigualdades existentes[4] y que afecta de manera desproporcionada a las poblaciones desfavorecidas, tener sistemas de salud con fondos insuficientes puede hacer a los países aún más vulnerables a los nuevos desafíos que el cambio climático plantea. Para los países de ALC, esto se traduce más probablemente en prestaciones e infraestructura de servicios de salud aún más deficientes, poniendo en riesgo la salud pública y el logro de los Objetivos de Desarrollo Sostenible (ODS). Dentro y entre los países de ALC se observan cifras contrastantes en la capacidad, preparación, respuesta y asignación de recursos de sus sectores de salud.

Por ejemplo, el gasto en salud ha crecido en ALC,[25] pero la distribución es muy desigual. Mientras países como Argentina, Brasil, Chile, Cuba y Uruguay gastan más del 9% de su producto interno bruto (PIB) en salud, Venezuela gasta menos del 4%.[26] Además, el crecimiento general del gasto no se ha traducido necesariamente en sistemas de salud más sostenibles y resilientes, e incluso donde existen esfuerzos deliberados para promover tal transición, los recursos asignados a ello varían mucho. Por ejemplo, mientras en 2019-2020 Uruguay gastó US$5,61 per cápita en adaptación climática para la salud y actividades relacionadas con la salud, Honduras gastó US$0,71.[4] Estas cifras muestran las disparidades dentro de ALC en su capacidad para responder y adaptarse a un clima cambiante, colocando a muchos países en un punto de partida desventajoso.

La región de ALC es un claro ejemplo de la creciente desigualdad entre la geografía de las emisiones y la geografía de los impactos. Mientras en 2018 los países de ALC generaron 2,6 toneladas métricas de emisiones de dióxido de carbono (CO_2) per cápita, los países de altos ingresos emitieron 10,3 toneladas métricas de emisiones de CO_2.[27] Mientras tanto, entre 1998 y 2020, los eventos climáticos han afectado a 277 millones de personas y provocado la muerte de más de 300.000 personas en la región de ALC.[28]

En términos de peligros climáticos, el informe de Estado del Clima en América Latina y el Caribe de la Organización Meteorológica Mundial[28] y el informe del Grupo de Trabajo I del IPCC como pate de su Sexto Informe de Evaluación[29,30] muestran cambios actuales y futuros en el sistema climático en comparación con los niveles preindustriales. Estos cambios incluyen ciclones tropicales extremos, tormentas y tormentas de polvo; sequías severas y extensas; tendencias crecientes de la temperatura media a tasas mayores que el promedio global que conducen a la pérdida de glaciares y a olas de calor; variaciones en precipitación que pueden ser aceleradas por las actuales tasas de cambio regional de uso del suelo y de deforestación; continuidad en el aumento relativo del nivel del mar; y un aumento de las olas de calor marinas.

El informe de *Lancet* Countdown envía un claro mensaje de alerta con un *"código rojo para la salud"*, destacando un aumento en la probabilidad de transmisión del dengue, el zika y el chikungunya por *Aedes aegypti* en Brasil y Perú.[4] Los casos de dengue casi se han triplicado, desde 2000-2009 (6,78 millones) a 2010-2019 (16,52 millones), donde el mayor registro de casos ocurrió en 2019.[31,32] Además, la exposición a olas de calor también ha aumentado en la región, alcanzando en 2020 casi 270 millones de personas-días en poblaciones vulnerables (>65 años).[4] Se estima que en Sudamérica entre el 20% y el 60% de la mortalidad relacionada con el calor se debió al cambio climático antropogénico entre 1991-2018.[33] Finalmente, el consumo excesivo de carnes rojas tiene tres consecuencias: cambio de uso del suelo para la producción ganadera, emisiones de GEI, especialmente metano, y aumento de la mortalidad prematura. *Lancet* Countdown informa, en 2018, la abrumadora cifra de 66.000 muertes prematuras y prevenibles atribuibles al consumo de carnes rojas, solo en Argentina, Brasil y Colombia.[4]

El cambio climático también puede conducir al desplazamiento forzado, a la migración y a la movilidad de la población, junto con pérdidas de vidas y el impacto económico que ello conlleva. Los impactos económicos de los eventos meteorológicos extremos en relación con el tamaño de la economía son singulares para la región del Caribe,[34] donde además se añade la amenaza significativa que representa el aumento del nivel del mar. Se ha estimado que el costo económico del huracán María en Dominica fue del 260% de su PIB anual, junto con un desplazamiento de hasta el 27,3% de su población total.[34] Miles de personas de estas islas también corren el riesgo de exposición al aumento del nivel del mar en el futuro. Según estimaciones proyectadas, solo en Haití 100 mil personas corren el riesgo de exponerse a un aumento futuro de 1 metro en el nivel del mar para fines de siglo[4].

3. Transformación de desafíos en oportunidades para mejorar la salud de las personas

Los desafíos antes mencionados comparten muchos impulsores inducidos por el ser humano, que pueden transformarse en oportunidades. Los países de ALC pueden optar por responder a la coyuntura crítica actual con una visión integral y, a través de una aún mayor coherencia de políticas intersectoriales, promover simultáneamente mejores sistemas de salud pública, acciones económicas dirigidas a la reducción de las desigualdades, proteger el medio ambiente y los servicios ecosistémicos, gestionar los riesgos climáticos y, en última instancia, mejorar la salud de las personas. La acción climática, tanto la mitigación como la adaptación, debe estar en el centro de una transición amplia y profunda hacia modelos de desarrollo más inclusivos,

justos y sostenibles, particularmente considerando que los beneficios económicos para la salud por hacer frente al cambio climático superan sustancialmente los costos de lograrlos.[14]

Los países de ALC son heterogéneos en clima, ecosistemas, población humana y cultura, pero comparten vulnerabilidades similares, reflejadas en una baja capacidad de adaptación, especialmente de los sistemas de salud.[14] Una óptica territorial de los riesgos para la salud comunitaria centrada en género, equidad y poblaciones vulnerables debe ser la prioridad para la construcción de resiliencia y la adaptación.[36] El último informe de *Lancet* Countdown muestra que las ciudades de ALC están esforzándose en evaluar los riesgos del cambio climático que enfrentan a nivel de ciudad. De las 250 ciudades encuestadas en ALC, un total de 182 tienen, en curso o realizado, un plan de evaluación de riesgo y vulnerabilidad al cambio climático.[4] Esto refleja un paso importante hacia la adaptación al cambio climático. La adecuada implementación de planes diseñados en función de vulnerabilidades específicas asociadas a las características sociales y ambientales de cada territorio, incorporando simultáneamente los sistemas de salud, ofrece una inmensa oportunidad para proteger la salud de las personas.

Complementariamente, el éxito de la mitigación del cambio climático es clave, ya que puede proporcionar, a nivel local, co-beneficios en salud. Considerando que se prevé un aumento de las emisiones y que hasta el 15% de las tasas de mortalidad por COVID-19 se han atribuido a la exposición a largo plazo a la contaminación del aire,[37] una economía descarbonizada es esencial para alcanzar los objetivos del Acuerdo de París y mejorar la salud. Algunos esfuerzos de descarbonización en la región son elogiables. Chile es el primer país de la región en introducir un impuesto al carbono[4] y su generación de electricidad a partir de emisiones de fuentes bajas en carbono ha aumentado en los últimos años, desde el 0% en 2007 al 14% en 2019, lo que sitúa a Chile por encima del promedio de los países de la OCDE.[38] Además, México está clasificado dentro de los 50 países del mundo con la mayor producción de electricidad a partir de fuentes renovables.[4] Las políticas vinculadas al transporte sostenible y a la eliminación gradual de los subsidios a los combustibles fósiles podrían ayudar a construir ciudades más limpias y sistemas de transporte que fomenten la actividad física, así como mejorar la salud al reducir la exposición a la contaminación del aire.

Los espacios verdes en las ciudades secuestran carbono y proveen enfriamiento local que interrumpe las islas de calor urbanas, lo que beneficia tanto a la mitigación del cambio climático como a la adaptación al calor. Según el informe de *Lancet* Countdown del año 2021, hay menos ciudades con espacios urbanos verdes en ALC que en el resto del mundo (27%). Por ejemplo, Lima es una de las seis capitales con menos espacios verdes en el mundo.[4] Invertir en espacios verdes es una gran oportunidad para las ciudades de ALC, ya que reduce la contaminación acústica y del aire, reduce el efecto isla de calor, alivia el estrés, promueve la actividad física y reduce la mortalidad general.[4,38]

Avanzar en sistemas alimentarios saludables, sostenibles y equitativos representa otras oportunidades para promover la buena salud para todas las personas, mientras se reducen las emisiones de GEI. El Programa Nacional de Alimentación Escolar de Brasil (PNAE, por su sigla en portugués) es una política sostenible innovadora que, en el marco del Programa de Cooperación Internacional Brasil-FAO,[39] estipula por ley que se gastará una cuota mínima del

30% de los fondos del PNAE en la adquisición de productos orgánicos provenientes de granjas familiares locales. Esta iniciativa no solo brinda acceso a una dieta más saludable, sino que también promueve la agricultura sostenible, fomenta el crecimiento económico local y reduce las emisiones de GEI de la cadena de suministro de alimentos.[40]

Todo lo anterior solo se puede lograr movilizando una cantidad significativa de financiamiento climático, que es fundamental para implementar planes de mitigación y adaptación. La Agenda de Acción de Addis Abeba sobre Financiamiento para el Desarrollo postula que los recursos públicos nacionales, complementados con asistencia internacional, según corresponda, son fundamentales para lograr el desarrollo sostenible.[41] En ese contexto, los países de ALC deben ser responsables de la implementación de sus propias NDC y Planes Nacionales de Adaptación (NAP, por su sigla en inglés), a través de compromisos financieros tangibles y transparentes que se traduzcan en asignaciones presupuestarias reales, y de establecer mecanismos de monitoreo y evaluación que permitan la rendición de cuentas y la evaluación del progreso.

Sin embargo, los países de ALC requerirán un significativo apoyo internacional si quieren adoptar objetivos y planes climáticos verdaderamente ambiciosos y transformadores. Vale la pena señalar que los países de la región, junto con otros países de ingresos bajos y medianos de todo el mundo, por mucho tiempo han visto incumplida la promesa de financiamiento por parte de las naciones industrializadas.[42] Según se declara en el Acuerdo de París, especialmente lo dispuesto en el Artículo 9,[43] los países industrializados deben movilizar USD$100 mil millones por año para financiar la acción climática en el Sur Global, un compromiso que sigue sin materializarse. Además, deben promover esfuerzos para hacer más accesibles los mecanismos financieros internacionales para los países en desarrollo.[42]

Por ejemplo, el Fondo Verde para el Clima (GCF, por su sigla en inglés) y el Fondo para el Medio Ambiente Mundial (GEF, por su sigla en inglés) tienen requisitos muy complejos que dificultan notablemente el acceso a sus recursos, y que la mayoría de los países de ALC no tienen la capacidad de encarar (para ilustrar, a octubre de 2021, solo 17 instituciones de la región operan como entidades acreditadas para implementar proyectos del GCF).[44] Además, el acceso al financiamiento internacional es especialmente difícil para los países de ALC porque la mayoría de ellos están clasificados como de ingresos medios (una clasificación basada únicamente en el PIB), y por lo tanto, no son priorizados para la cooperación internacional, mientras que aquellos que se han “graduado” ya no son elegibles para recibir asistencia oficial para el desarrollo.[45] Esto se complica aún más por la crisis extrema de deuda que está sumergiendo a muchos países de la región, particularmente en el Caribe.

En este contexto, es más crucial que nunca que los países desarrollados apoyen las iniciativas internacionales que tienen como objetivo abordar simultáneamente las crisis sanitaria y climática, tales como el Programa de salud de la COP26,[46] y financiar integralmente las acciones para prevenir y abordar las pérdidas y los daños causados por los impactos climáticos, particularmente a través del Mecanismo Internacional de Varsovia y la Red de Santiago sobre Pérdidas y Daños.[47] Para que ALC esté en condiciones de responder de manera decisiva y transformadora a los riesgos climáticos y de salud que se refuerzan mutuamente, es esencial no solo asegurar los medios de implementación, sino facilitar el acceso regional efectivo a los mismos. No obstante, frente a la convergencia de la crisis de la pandemia de COVID-19 y la

emergencia climática —y para comprender los impulsores climáticos y ambientales de las enfermedades emergentes y reemergentes, para poder anticipar, prevenir, preparar, responder y recuperarse— todos los países deben actuar, sin importar su nivel de desarrollo, y las economías más grandes de la región de ALC están llamadas a modelar con el ejemplo. Desafortunadamente, hasta ahora este no ha sido el caso. Según el programa de recuperación verde de la Organización de las Naciones Unidas (ONU), el gasto en rescate y recuperación ambientalmente sostenible en ALC es imperceptible.[48] Los países deben aprovechar la oportunidad para estructurar su recuperación económica del COVID-19 en torno a medidas ambientalmente inteligentes que construyan un futuro más sostenible. Esto puede tener un efecto multiplicador con la creación de empleos verdes, la reducción de emisiones, el aumento de la resiliencia y, en última instancia, el crecimiento económico.[49]

4. El sector salud como actor clave en la acción climática

Las y los profesionales de la salud tienen una voz confiable que puede influir en la salud y el bienestar de las personas en el contexto de la crisis climática.[50,51] El sector salud se convierte en un actor clave en lo que respecta a la incidencia, la articulación de la necesidad de acción climática y al establecimiento de puentes con otros actores, favoreciendo la acción intersectorial a través de la perspectiva de los co-beneficios en salud. Las y los profesionales de la salud refuerzan cada vez más su compromiso con el tema — desde trabajar con las comunidades afectadas por el cambio climático hasta asumir la defensa del clima y trabajar para reducir las emisiones de los sistemas de salud.[50,52] Las personas que ejercen las profesiones de la salud y las ciencias climáticas son voces confiables en la mayoría de las sociedades y, por lo tanto, pueden ser campeonas para ayudar a diseñar políticas climáticas que mejoren los resultados de salud y el bienestar humano.

Los sistemas de salud tienen una huella ambiental significativa. Organizaciones como Salud sin Daño estimaron que en 2014 la huella de carbono del sector representó el 4,4% de las emisiones globales netas de GEI[53,54] con un aumento hasta el 4,9% según el último informe de *Lancet* Countdown.[4] Los establecimientos de salud resilientes al clima y ambientalmente sostenibles contribuyen a una alta calidad de atención y accesibilidad de los servicios y, al ayudar a reducir los costos, también aseguran una mejor asequibilidad.[55,56] El sector puede fortalecer los sistemas de salud en general y mejorar los establecimientos de atención en salud, que son la primera línea de defensa en caso de eventos extremos y *shocks* climáticos.[55] La construcción de sistemas de salud resilientes al clima se ha vuelto fundamental para cualquier estrategia nacional de adaptación, especialmente a través de medidas de adaptación que al mismo tiempo impulsen la equidad en salud. Las herramientas existentes que evalúan las vulnerabilidades en los establecimientos de salud[57], que calculan y monitorean la huella de carbono,[58] y los documentos de orientación para construir sistemas de salud resilientes al clima,[59] pueden ayudar al sector a estar mejor preparado en el contexto del cambio climático.

Los gobiernos también pueden lograr tanto la resiliencia de la salud como los ODS (*i*) invirtiendo en sectores determinantes de la salud; (*ii*) priorizando el logro de una Cobertura Sanitaria Universal y de una vida sana y bienestar (ODS3) como un objetivo político dominante; (*iii*) asegurando la coordinación entre sectores a través de comités interministeriales

sobre cambio climático, que incluyan al sector salud; y (*iv*) monitoreando las sinergias y compensaciones resultado de las acciones de sectores determinantes de la salud,[55] como los sectores de energía, transporte, alimentación y vivienda. Un ejemplo de cómo los sistemas de salud pueden adaptarse mejor al cambio climático y desarrollar resiliencia es con los sistemas de alerta temprana de olas de calor, donde los servicios meteorológicos nacionales se articulan con los sistemas de salud. Según el informe de *Lancet* Countdown del año 2021, solo cinco de los 179 países miembros de la OMS que respondieron indicaron que los servicios climáticos nacionales guían sus políticas y planes de inversión en el sector; como era de esperar, ninguno es de ALC.[4]

Una forma de asegurar que exista coherencia en las políticas para el clima y la salud es garantizar un mandato explícito y de alto nivel para la implementación integrada con mecanismos de control y evaluación. Un estándar clave es incluir la salud en las NDC. Por ejemplo, la NDC de Costa Rica es la única en la región alineada con el Acuerdo de París de acuerdo a la evaluación elaborada por GCHA sobre NDC saludables.[60,61] Dicha NDC reconoce los impactos del cambio climático en la salud: enfermedades transmitidas por vectores y seguridad alimentaria, por mencionar algunos, y se compromete a fortalecer el conocimiento, la capacidad de monitoreo y sus servicios de respuesta de vigilancia de la salud.[62] La salud se considera en la adaptación y las finanzas, y en los sectores de transporte y energía se da énfasis a los beneficios para la salud de objetivos de mitigación específicos, con límites de tiempo. Otro ejemplo es Argentina, que fue el primer país en incluir medidas de mitigación del sector salud en sus NDC, y ha incluido la salud como un "eje rector" que sustenta gran parte del contenido de sus NDC.[63] La Organización Panamericana de la Salud/Organización Mundial de la Salud (OMS) ha desarrollado sugerencias y ejemplos adicionales sobre cómo elaborar NDC inclusivas de la salud.[64] Además, este mandato de alto nivel también puede ser adoptado por los sistemas y establecimientos de salud. Por ejemplo, 19 hospitales e instituciones médicas de la región se han sumado al componente de salud de la campaña Carrera hacia el Cero de la ONU.[65]

El sector salud está incluso convirtiéndose en un actor en las negociaciones climáticas internacionales como quedó de manifiesto con la participación, sin precedentes, de profesionales de la salud durante la 26ª Conferencia de las Naciones Unidas sobre el Cambio Climático (COP26), en Glasgow. Más de 50 países de todo el mundo se unieron al Programa de salud de la COP26,[46] que hizo un llamado a los países a presentar compromisos nacionales para desarrollar (*i*) sistemas de salud resilientes al clima y (*ii*) sistemas de salud sostenibles y bajos en emisiones.[66] Los países de ALC signatarios incluyeron a Argentina, Bahamas, Belice, Chile, Colombia, Costa Rica, Jamaica, Panamá, Perú y República Dominicana.[67] Estos logros podrían interpretarse como señales de progreso.

En la COP del año pasado, la Presidencia del Reino Unido organizó un evento de alto nivel centrado en la salud, donde la comunidad global de la salud destacó las 10 recomendaciones del Informe Especial de la OMS para la COP *El argumento de salud para la acción climática.*[55] Además, 46 millones de profesionales de la salud enviaron una Carta de Prescripción para un Clima Saludable[68] a las y los líderes mundiales en la COP26, pidiendo que la salud y la equidad se coloquen en el centro de la respuesta climática. La carta fue entregada formalmente en el

evento de la Presidencia y cientos de profesionales de la salud de 12 países de ALC la firmaron, en apoyo a la iniciativa global. Panamá reforzó la voz de la comunidad mundial de la salud en el plenario al expresar su insatisfacción por el hecho de que las decisiones finales de la COP26 no reconocieron la importancia de comprender y abordar los impactos del cambio climático en la salud y los beneficios de la acción climática para la salud.[69]

5. Conclusiones

La evidencia actual muestra que las tendencias climáticas críticas están empeorando, exacerbando la pobreza y la desigualdad debido a las respuestas tardías e inconstantes de todos los países del mundo; los países de ALC no son una excepción. Sin embargo, incluso dentro de la región de ALC y dentro de cada país, las poblaciones menos responsables de la situación serán las más afectadas. Además, si no se prioriza la equidad, ellas también serán las últimas en disfrutar de los co-beneficios de la adaptación y de los acelerados esfuerzos de descarbonización.

Los gobiernos de ALC necesitan acciones políticas urgentes para fortalecer el sistema de salud y asegurar que la salud esté en el centro de la respuesta nacional y regional a la crisis climática. El sector salud debe convertirse en la intersección articuladora de otras partes interesadas y favorecer la acción intersectorial a través de la perspectiva de los co-beneficios en salud. Los países deben fortalecer el componente de salud de sus instrumentos de políticas climáticas, en particular las NDC y los Planes Nacionales de Adaptación. Asimismo, ALC debe inspirarse en las experiencias exitosas de los países vecinos para promover la gobernanza ambiental y de la salud.

Además, la comprensión de las vulnerabilidades al cambio climático relacionadas con la salud individual de los países de ALC apoyará la identificación de acciones prácticas, adaptadas a las dificultades geográficas específicas, para construir sistemas de salud resilientes. A esta complejidad debemos agregar el desafío adicional de capacitar a las y los profesionales de la salud para que sean capaces de considerar los impactos del cambio climático en sus planes de diagnóstico y tratamiento. En la mayoría de las sociedades, son una voz confiable que está presente, como ninguna otra profesión, en las zonas más remotas de los países de ALC. Así, el compromiso climático debe contemplar el desarrollo de capacidades de profesionales de la salud, la asignación de recursos suficientes y la ampliación de los servicios de salud, así como de marcos de monitoreo y evaluación.

Como personas ciudadanas y voceras de la ciencia, hacemos un llamado a los gobiernos para que intensifiquen la ambición climática, reforzando las medidas de adaptación y mitigación, centrándose en la salud y la equidad, y acelerando el desfasamiento de los combustibles fósiles garantizando al mismo tiempo una transición justa. Desde un espacio de ciencia e incidencia, la *Red de Clima y Salud de América Latina y el Caribe* ofrece colaboración en investigación, evaluación e incidencia a gobiernos nacionales y a organizaciones multilaterales y no gubernamentales para aprovechar la oportunidad de mejorar la salud y el bienestar de las generaciones actuales y futuras.

Colaboradores/as: MYG, MS, AHE y SH conceptualizaron el artículo de opinión; MYG, MS, AHE y YP escribieron el primer borrador; SC, DB y SH realizaron revisiones críticas del manuscrito y contribuyeron a la redacción; todos los autores revisaron y acordaron la versión final de la perspectiva. La Red de Clima y Salud de América Latina y el Caribe está de acuerdo con el contenido de la perspectiva.

Reconocimientos: SH y MYG recibieron fondos de una subvención sin restricciones de Wellcome Trust (209734/Z/17/Z). Agradecemos a la Dra. Jeni Miller (Alianza Global para el Clima y la Salud) quien brindó información y experiencia que mejoraron enormemente el manuscrito.

Declaración de intereses: Las autoras declaran que no existe conflicto de interés. Las autoras son las únicas responsables de las opiniones expresadas en el manuscrito, que pueden no necesariamente reflejar la opinión o la política de la OPS.

Referencias

1 World Bank. Disaster Risk Management in Latin America and the Caribbean Region: GFDRR Country Notes. Washington, DC: World Bank, 2012 https://openknowledge.worldbank.org/handle/10986/27336 (accessed Oct 20, 2021).

2 Economic Commission for Latin America and the Caribbean. Report on the economic impact of coronavirus disease (COVID-19) on Latin America and the Caribbean. CEPAL, 2020 https://www.cepal.org/en/publications/45603-report-economic-impact-coronavirus-disease-covid-19-latin-america-and-caribbean (accessed Oct 20, 2021).

3 Economic Commission for Latin America and the Caribbean. Social Panorama of Latin America. Santiago, 2021 https://repositorio.cepal.org/bitstream/handle/11362/46688/8/S2100149_en.pdf.

4 Romanello M, McGushin A, Napoli CD, *et al.* The 2021 report of the Lancet Countdown on health and climate change: code red for a healthy future. *The Lancet* 2021; **0**. DOI:10.1016/S0140-6736(21)01787-6.

5 Intergovernmental Panel on Climate Change. IPCC AR6-WGI Atlas [Internet]. IPCC WGI Interactive Atlas: Regional information (Advanced). 2021 [cited 2021 Oct 20]. Available from: https://interactive-atlas.ipcc.ch/atlas

6 Steffen W, Richardson K, Rockström J, *et al.* Planetary boundaries: Guiding human development on a changing planet. *Science* 2015; **347**: 1259855.

7 World Resources Institute. Key Visualizations | Climate Watch. 2016. https://www.climatewatchdata.org/key-visualizations?geographies=global&tags=ghg_emissions&topic=greenhouse_gases (accessed Oct 21, 2021).

8 Bárcena A, Samaniego J, Galindo LM, Ferrer J, Alatorre JE, Stockins P, et al. Economics of climate change in Latin America and the Caribbean [Internet]. 2014 p. 1. Available from:

https://www.cepal.org/sites/default/files/infographic/files/infographic_economics_of_climate_change.pdf

9 Climate Action Tracker. Climate Action Tracker [Internet]. Find your country. [cited 2022 Jan 11]. Available from: https://climateactiontracker.org/

10 The Global Climate and Health Alliance. Healthy NDCs. The Global Climate and Health Alliance. 2021. https://climateandhealthalliance.org/initiatives/healthy-ndcs/ (accessed Oct 20, 2021).

11 NDC Partnership. "NDC-RD 2020": The Dominican Republic's Widely Embraced Climate Plan |. 2020. https://ndcpartnership.org/news/%E2%80%9Cndc-rd-2020%E2%80%9D-dominican-republic%E2%80%99s-widely-embraced-climate-plan (accessed Jan 11, 2022).

12 Caribe CE para AL y el. Salud y cambio climático: metodologías y políticas públicas. CEPAL, 2021 https://www.cepal.org/es/publicaciones/47534-salud-cambio-climatico-metodologias-politicas-publicas (accessed Jan 11, 2022).

13 World Health Organization. 2021 WHO Health and Climate Change Survey Report. 2021. https://www.who.int/publications-detail-redirect/9789240038509 (accessed March 1, 2022).

14 World Health Organization. COP24 Special report: Health & Climate Change. 2018.

15 Welch C. First study of all Amazon greenhouse gases suggests the damaged forest is now worsening climate change [Internet]. Environment News. 2021 [cited 2022 Jan 11]. Available from: https://www.nationalgeographic.com/environment/article/amazon-rainforest-now-appears-to-be-contributing-to-climate-change

16 Gatti LV, Basso LS, Miller JB, *et al.* Amazonia as a carbon source linked to deforestation and climate change. *Nature* 2021; **595**: 388–93.

17 Pereira JC, Viola E. Brazilian climate policy (1992–2019): an exercise in strategic diplomatic failure. *Contemporary Politics* 2021; **0**: 1–24.

18 Hochstetler K, Viola E. Brazil and the politics of climate change: beyond the global commons. *Environmental Politics* 2012; **21**: 753–71.

19 World Wildlife Fund. Brazilian NDC reduces the country's climate ambition. 2020. https://wwf.panda.org/wwf_news/?1173241/WWF-Brazil-NDC (accessed Jan 12, 2022).

20 World Health Organization. Operational framework for building climate resilient health systems. 2015. https://www.who.int/publications-detail-redirect/operational-framework-for-building-climate-resilient-health-systems (accessed Feb 28, 2022).

21 Kronik J, Verner D. Indigenous Peoples and Climate Change in Latin America and the Caribbean [Internet]. Washington D.C: The World Bank; 2010 p. 208. Available from: https://openknowledge.worldbank.org/bitstream/handle/10986/2472/555400PUB0Indi1EPI1958810601PUBLIC1.pdf?sequence=1&isAllowed=y

22 FAO and FILAC. Forest governance by indigenous and tribal peoples: An opportunity for climate action in Latin America and the Caribbean. Santiago, Chile: FAO, 2021 https://www.fao.org/documents/card/en/c/cb2930en (accessed March 1, 2022).

23 Transparency International. Corruption Perception Index 2021 [Internet]. 2021 [cited 2022 Feb 24] p. 22. Available from: https://images.transparencycdn.org/images/CPI2021_Report_EN-web.pdf

24 Writer NRHS. Harvard panel discusses protests across Latin America. Harvard Gazette. 2021; published online Nov 8. https://news.harvard.edu/gazette/story/2021/11/harvard-panel-discusses-protests-across-latin-america/ (accessed Jan 11, 2022).

25 OECD, The World Bank. Health at a Glance: Latin America and the Caribbean 2020. OECD, 2020 DOI:10.1787/6089164f-en.

26 World Bank. Current health expenditure (% of GDP) - Latin America & Caribbean | Data. 2018. https://data.worldbank.org/indicator/SH.XPD.CHEX.GD.ZS?locations=ZJ (accessed Oct 20, 2021).

27 World Bank. CO2 emissions (metric tons per capita) | Data. https://data.worldbank.org/indicator/EN.ATM.CO2E.PC (accessed Jan 11, 2022).

28 World Meteorological Organization (WMO). State of the Climate in Latin America and the Caribbean 2020 (WMO-No. 1272). Geneva: WMO, 2021.

29 Intergovernmental Panel on Climate Change. Regional fact sheet – Central and South America [Internet]. IPCC; [cited 2021 Oct 20] p. 2. (Working Group I – The Physical Science Basis). Report No.: Sixth Assessment Report. Available from: https://www.ipcc.ch/report/ar6/wg1/downloads/factsheets/IPCC_AR6_WGI_Regional_Fact_Sheet_Central_and_South_America.pdf

30 Intergovernmental Panel on Climate Change. Regional fact sheet - North and Central America. Sixth Assessment Report. Working Group 1 - The Physcal Science Basis. https://www.ipcc.ch/report/ar6/wg1/downloads/factsheets/IPCC_AR6_WGI_Regional_Fact_Sheet_North_and_Central_America.pdf (accessed Oct 20, 2021).

31 World Health Organization. PAHO/WHO Data - Dengue. https://www3.paho.org/data/index.php/en/mnu-topics/indicadores-dengue-en.html (accessed Jan 12, 2022).

32 World Health Organization. Epidemiological Update: Dengue - 7 February 2020 - PAHO/WHO | Pan American Health Organization. 2020. https://www.paho.org/en/documents/epidemiological-update-dengue-7-february-2020 (accessed Jan 12, 2022).

33 Vicedo-Cabrera AM, Scovronick N, Sera F, *et al.* The burden of heat-related mortality attributable to recent human-induced climate change. *Nature Climate Change* 2021; **11**: 492–500.

34 Economic Commission for Latin America and the Caribbean, Bárcena A, Samaniego J, Peres Núñez W, Alatorre JE. La emergencia del cambio climático en América Latina y el

Caribe: ¿seguimos esperando la catástrofe o pasamos a la acción? UN, 2020 DOI:10.18356/1f198404-es.

35 Economic Commission for Latin America and the Caribbean. The outlook for oceans, seas and marine resources in Latin America and the Caribbean: Conservation, sustainable development and climate change mitigation. 2020; : 77.

36 Miranda-Chacón Z, Chavarría ALR, Troyo A, Rivera-Bermúdez G, Hidalgo HG, Alfaro EJ. The Lancet Countdown on Health and Climate Change: Policy brief for Costa Rica [Internet]. Costa Rica: Lancet Countdown South America; 2021 [cited 2021 Oct 20] p. 7. Available from: https://www.dropbox.com/s/6pjl9xcjppnit8s/Costa%20Rica%202021%20-%20Lancet%20Countdown%20Policy%20Brief.pdf?dl=0

37 Economic Commission for Latin America and the Caribbean. La paradoja de la recuperación en América Latina y el Caribe. Crecimiento con persistentes problemas estructurales: desigualdad, pobreza, poca inversión y baja productividad. 2021; : 42.

38 Palmeiro-Silva Y, Reyes Paecke S, Sauma Santis E, Moya Osorio J. Chile 2021 - Lancet Countdown Policy Brief. The Lancet Countdown, 2021 https://www.dropbox.com/s/ux2ypm16ej8nrrb/Chile%202021%20-%20Lancet%20Countdown%20Policy%20Brief.pdf?dl=0.

39 Food and Agriculture Organization of the United Natins. Learning from Sustainable School Feeding in Brazil. 2017. https://www.fao.org/americas/noticias/ver/en/c/1043379/ (accessed Jan 11, 2022).

40 Fundo Nacional de Desenvolvimento da Educação. Sobre o Pnae [Internet]. Programa Nacional de Alimentação Escolar. [cited 2022 Jan 11]. Available from: https://www.fnde.gov.br/index.php/programas/pnae/pnae-sobre-o-programa/pnae-sobre-o-pnae

41 United Nations. Addis Ababa Action Agenda of the Third International Conference on Financing for Development. 2015.

42 Timperley J. The broken $100-billion promise of climate finance — and how to fix it. *Nature* 2021; **598**: 400–2.

43 United Nations Framework Convention on Climate Change. Adoption of the Paris Agreement [Internet]. 2015 [cited 2022 Feb 25]. Available from: https://unfccc.int/sites/default/files/english_paris_agreement.pdf

44 Samaniego J, Schneider H. Cuarto informe sobre financiamiento para el cambio climático en América Latina y el Caribe, 2013-2016 [Internet]. Santiago: Comisión Económica para América Latina y el Caribe (CEPAL; 2019 [cited 2022 Feb 25] p. 112. Report No.: Cuarto informe. Available from: https://repositorio.cepal.org/bitstream/handle/11362/44487/1/S1900399_es.pdf

45 Economic Commission for Latin America and the Caribbean. It is Urgent that the International Community Support the End of Middle-Income Countries' Graduation from Official Development Assistance. 2021; published online July 5.

https://www.cepal.org/en/news/it-urgent-international-community-support-end-middle-income-countries-graduation-official (accessed Jan 11, 2022).

46 World Health Organization. COP26 Health Programme. WHO, COP26 Presidency, HCWH, 2021.

47 United Nations Framework Convention on Climate Change. Warsaw International Mechanism for Loss and Damage associated with Climate Change Impacts (WIM) [Internet]. United Nations Climate Change. [cited 2022 Jan 11]. Available from: https://unfccc.int/topics/adaptation-and-resilience/workstreams/loss-and-damage/warsaw-international-mechanism

48 United Nations Environment Programme. State of play in Latin America and the Caribbean. Recuperación Verde. 2021; published online Dec 22. https://recuperacionverde.com/en/state-of-play-in-latin-america-and-the-caribbean-may-september-2021/ (accessed Feb 25, 2022).

49 Organisation for Economic Co-operation and Development. Policy Brief: Making the green recovery work for jobs, income and growth - OECD. https://read.oecd-ilibrary.org/view/?ref=136_136201-ctwt8p7qs5&title=Making-the-Green-Recovery-Work-for-Jobs-Income-and-Growth_ (accessed Feb 25, 2022).

50 Healthcare Without Harm. Health professionals are both trusted communicators and important actors when it comes to protecting public health [Internet]. Health Professional Advocates The movement for healthy people and a healthy planet. [cited 2021 Nov 5]. Available from: https://healthcareclimateaction.org/professionals

51 Maibach E, Frumkin H, Ahdoot S. Health Professionals and the Climate Crisis: Trusted Voices, Essential Roles. *World Medical & Health Policy* 2021; **13**: 137–45.

52 Kotcher J, Maibach E, Miller J, *et al.* Views of health professionals on climate change and health: a multinational survey study. *The Lancet Planetary Health* 2021; **5**: e316–23.

53 Pichler P-P, Jaccard IS, Weisz U, Weisz H. International comparison of health care carbon footprints. *Environ Res Lett* 2019; **14**: 064004.

54 Karliner J, Slotterback S. Health care´s climate footprint: How the health sector contributes to the global climate crisis and opportunities for action [Internet]. Health Care Without Harm; 2019 [cited 2022 Mar 1]. Available from: https://noharm-global.org/sites/default/files/documents-files/5961/HealthCaresClimateFootprint_092319.pdf

55 World Health Organization. The Health Argument for Climate Action. WHO, 2021.

56 Karliner J, Roschnik S. Global Road Map for Health Care Decarbonization. A navigational tool for achieving zero emissions with climate resilience and health equity [Internet]. Healthcare Without Harm; 2021 [cited 2021 Oct 21]. Available from: https://healthcareclimateaction.org/sites/default/files/2021-08/Global%20Road%20Map%20for%20Health%20Care%20Decarbonization.pdf

57 World Health Organization. Checklists to Assess vulnerabilities in Health Care Facilities in the Context of Climate Change. 2021. https://www.who.int/publications-detail-

redirect/checklists-vulnerabilities-health-care-facilities-climate-change (accessed Jan 11, 2022).

58 Healthcare Without Harm. Herramienta para hospitales y sistemas de salud [Internet]. Monitoreo del impacto climático. 2022 [cited 2022 Jan 11]. Available from: https://saludsindanio.org/monitoreo

59 World Health Organization. WHO guidance for climate resilient and environmentally sustainable health care facilities. https://www.who.int/publications-detail-redirect/9789240012226 (accessed Jan 11, 2022).

60 The Global Climate and Health Alliance. NDC Scorecards. The Global Climate and Health Alliance. https://climateandhealthalliance.org/initiatives/healthy-ndcs/ndc-scorecards/ (accessed Jan 11, 2022).

61 Dirección de Cambio Climático. Costa Rica lidera ranking internacional de políticas climáticas que benefician la salud pública. 2021; published online July 15. https://cambioclimatico.go.cr/costa-rica-lidera-ranking-internacional-politicas-climaticas-salud-publica/ (accessed Jan 11, 2022).

62 Ministerio de Ambiente y Energía. Contribución Nacionalmente Determinada (NDC) de Costa Rica. Dirección de Cambio Climático. https://cambioclimatico.go.cr/contribucion-nacionalmente-determinada-ndc-de-costa-rica/ (accessed Feb 25, 2022).

63 Ministerio de Ambiente y Desarrollo Sostenible de Argentina. Segunda Contribución Determinada a Nivel Nacional de la República Argentina [Internet]. 2020 [cited 2021 Nov 5]. Available from: https://www4.unfccc.int/sites/ndcstaging/PublishedDocuments/Argentina%20Second/Argentina_Segunda%20Contribuci%C3%B3n%20Nacional.pdf

64 World Health Organization. Enhancing health and climate change commitments in updated Nationally Determined Contributions. 2021.

65 Healthcare Without Harm. Race to Zero Partner [Internet]. Join the largest ever alliance committed to achieving net zero carbon emissions. 2021 [cited 2021 Oct 21]. Available from: https://healthcareclimateaction.org/racetozero

66 World Health Organization. Countries commit to develop climate-smart health care at COP26 UN climate conference. https://www.who.int/news/item/09-11-2021-countries-commit-to-develop-climate-smart-health-care-at-cop26-un-climate-conference (accessed Nov 9, 2021).

67 Posse CG. Diez países de América Latina y el Caribe se suman al Programa de salud de la COP26 [Internet]. Comunicado de prensa. 2021 [cited 2021 Nov 9]. Available from: https://saludsindanio.org/comunicado/programa-salud-COP26

68 The Global Climate and Health Alliance. Healthy Climate Prescription. #HealthyClimatePrescription. https://healthyclimateletter.net/ (accessed Jan 11, 2022).

69 Reuters. LIVE: COP President Alok Sharma holds informal stocktaking plenary [Internet]. Glasgow; 2021 [cited 2022 Jan 11]. Available from: https://www.youtube.com/watch?v=oL2l7LktV6k
